# Fully automated platelet differential interference contrast image analysis via deep learning

**DOI:** 10.1038/s41598-022-08613-2

**Published:** 2022-03-17

**Authors:** Carly Kempster, George Butler, Elina Kuznecova, Kirk A. Taylor, Neline Kriek, Gemma Little, Marcin A. Sowa, Tanya Sage, Louise J. Johnson, Jonathan M. Gibbins, Alice Y. Pollitt

**Affiliations:** 1 School of Biological Sciences, University of Reading, Reading, UK; 2 The Brady Urological Institute, Johns Hopkins School of Medicine, Baltimore, USA

**Keywords:** Computational models, Haematological diseases, Data acquisition

## Abstract

Platelets mediate arterial thrombosis, a leading cause of myocardial infarction and stroke. During injury, platelets adhere and spread over exposed subendothelial matrix substrates of the damaged blood vessel wall. The mechanisms which govern platelet activation and their interaction with a range of substrates are therefore regularly investigated using platelet spreading assays. These assays often use differential interference contrast (DIC) microscopy to assess platelet morphology and analysis performed using manual annotation. Here, a convolutional neural network (CNN) allowed fully automated analysis of platelet spreading assays captured by DIC microscopy. The CNN was trained using 120 generalised training images. Increasing the number of training images increases the mean average precision of the CNN. The CNN performance was compared to six manual annotators. Significant variation was observed between annotators, highlighting bias when manual analysis is performed. The CNN effectively analysed platelet morphology when platelets spread over a range of substrates (CRP-XL, vWF and fibrinogen), in the presence and absence of inhibitors (dasatinib, ibrutinib and PRT-060318) and agonist (thrombin), with results consistent in quantifying spread platelet area which is comparable to published literature. The application of a CNN enables, for the first time, automated analysis of platelet spreading assays captured by DIC microscopy.

## Introduction

Platelets are the main contributor to arterial thrombosis which is a leading cause of myocardial infarction and stroke^1,2^. During injury, platelets rapidly adhere and spread over exposed subendothelial matrix substrates of the damaged blood vessel wall to arrest bleeding and facilitate wound healing^2,3^. The mechanisms which govern platelet activation and their interaction with a range of substrates are therefore regularly assessed and investigated using platelet spreading assays. These assays allow biological processes such as platelet adhesion and morphology to be investigated^4,5^.

Differential interference contrast (DIC) microscopy is an optical imaging technique commonly used to quantify the behaviour of individual platelets within spreading assays; owing to its ability to enhance the contrast between the platelet and background. These DIC images can be used to measure individual platelet features such as spread area, perimeter, and circularity.

However, translating the information encoded within an image, such as the location of a platelet, into a quantitative value that can be used for down-stream analysis remains logistically challenging. Whilst DIC imaging enables the use of label free cells, it also creates an unwanted shadow artefact within the image^6^. In turn, this prevents the use of automated segmentation techniques commonly applied to epi-fluorescent images e.g., thresholding and edge detection. As a result, a process of manual segmentation is often used that is extremely time consuming as human input is required throughout. Furthermore, manual segmentation also introduces a high degree of user subjectivity and variability into the analytical workflow that may subsequently impact on the biological insight that is gained^7^. The introduction of deep learning approaches, such as convolutional neural networks (CNNs), offer an opportunity to overcome the limitations associated with DIC imaging and leverage the power of high-throughput single cell analysis^8,9^.

Automated segmentation has two stages: feature computation and feature selection^10^. Feature computation captures the information that is encoded in an image and translates it into a numerical value, such as the colour and intensity of a pixel or the length of an object^11^. Feature selection then builds a model from the extracted features that can be used to segment cells in future unseen images. The parameters for each feature in the model are estimated dependent on their discriminatory power, the higher the power the larger the weighting^12^. Yet, whilst a number of different automated segmentation approaches exist^13–15^, they typically all rely upon the same computed features that are defined a priori, for example the maximum area of a cell or the intensity of a pixel. In contrast, CNNs can achieve much higher levels of segmentation accuracy by using a data driven approach that deconstructs an image into multiple levels of abstraction^16^. Abstraction refers to the characterization of essential, but often unintuitive, features within an image that reduce the informational load and complexity. The abstractions combine low level features such as edges and curves with higher order features such as shapes to detect complex objects within an image, for example the morphology of a platelet^17^. As a result, the application of CNNs to automate DIC imaging offers an exciting opportunity to overcome a major bottleneck in the experimental workflow.

To produce accurate results, CNNs require large quantities of training data that can be potentially expensive and time consuming to curate. However, exactly how much data is needed during training remains poorly defined. Additionally, to fully leverage the power of a CNN, the trained model needs to be generalisable^18^. That is, the same trained model needs to be applicable across multiple different experimental conditions. In a biological setting, where data are typically collected in a sequential manner, a high degree of generalisability is essential to ensure that the model does not need to be retrained between each experiment.

Here, we demonstrate, for the first time, the application of a CNN to fully automate the analysis of platelet spreading assays captured by DIC microscopy. Next, we perform a quantitatively robust analysis of segmentation performance with respect to both the quantity and quality of the training data. Finally, we demonstrate the generalisability of the trained CNN when applied to extremes in platelet morphologies. In summary, we show that a CNN can be used to effectively analyse platelet morphology which abrogates time consuming manual analyses.

## Methods

### Washed platelet preparation

All protocols were approved by the University of Reading Research Ethics Committee and all experiments were performed in accordance with relevant guidelines and regulations. Informed consent was obtained from all blood donors prior to donation. Briefly, blood was drawn from healthy, drug free donors, from the antecubital fossa vein into sodium citrated (3.2%) vacutainers using a 21G butterfly needle. The first 3 mL of blood was taken into an EDTA vacutainer and discarded to avoid any tissue factor contamination^19^.

Acid-citrate-dextrose 10% (v/v) (ACD: 85 mM sodium citric acid, 111 mM glucose and 78 mM citric acid) was added to citrated whole blood prior to centrifugation at 200×*g* for 20 min, and the platelet rich plasma (PRP) harvested. Platelet sedimentation at 1000×*g*, for 10 min in the presence of 45 ng/mL prostacyclin (PGI_2_) preceded the removal of the plasma supernatant. The platelets were resuspended in modified Tyrode’s-HEPES buffer (134 mM NaCl, 2.9 mM KCl, 0.34 mM Na_2_HPO_4_·12H_2_0, 12 mM NaHCO_3_, 20 mM HEPES, 1 mM MgCl_2_ and 5 mM Glucose, pH 7.3), ACD (10% (v/v)) and 45 ng/mL PGI_2_ and centrifuged at 1000×*g*, for a further 10 min. Finally, the platelet pellet was resuspended to 4 × 10^8^ platelets/mL in Tyrode’s buffer and rested at 30 °C for 30 min. Platelets were diluted further to 1 × 10^7^ platelets/mL prior to platelet spreading assays.

### Mouse platelet preparation

All procedures were undertaken in accordance with a UK Home Office licence and approved by the University of Reading’s Animal Welfare and Ethical Review Body. Blood was drawn by cardiac puncture into 50 μL 3.2% sodium citrate following terminal CO_2_ narcosis. Whole blood was diluted using modified Tyrode’s-HEPES buffer and centrifuged at 200×*g* for 8 min. PRP was aspirated and centrifuged at 200×*g* for 2 min in the presence of 0.1 μg/mL PGI_2_. The supernatant was carefully aspirated avoiding the red cell pellet. Finally, platelets were pelleted at 1000×*g* for 5 min before resuspension in Tyrode’s buffer at 2 × 10^8^ platelets/mL and rested at 30 °C for 30 min. Platelets were diluted further to 1 × 10^7^ platelets/mL prior to platelet spreading assays.

### Platelet spreading assay

Glass coverslips were coated with either 100 μg/mL human fibrinogen (Sigma), 10 μg/mL Cross-linked collagen related peptide (CRP-XL), or 10 μg/mL von Willebrand factor (vWF) overnight at 4 °C. Unbound substrates were removed and coverslips washed × 3 using phosphate buffered saline (PBS: 10 mM Na_2_HPO_4_, 1.8 mM KH_2_PO_4_, 2.7 mM KCl and 137 mM NaCl, pH 7.4). Coverslips were blocked with 5 mg/mL heat denatured bovine serum albumin (BSA) dissolved in PBS for 45 min to avoid unspecific platelet attachment. Coverslips were washed × 3 with PBS before spreading 1 × 10^7^ platelets/mL washed platelets and incubated at 37 °C humidified atmosphere, 5% CO_2_ for 45 min. The time from venepuncture or cardiac puncture to platelet spreading remained constant at 1.5 and 1 h respectively, for all experiments. Following incubation, platelets were fixed using 10% formalin solution (Sigma) for 10 min, washed × 3 with PBS, and coverslips mounted onto glass slides using hydromount ready for imaging.

Platelets treated with inhibitors (Ibrutinib [1 μM], Dasatinib [10 μM] and PRT-060318 [5 μM]) and thrombin (0.1 U/mL) were incubated at 30 °C for 10 min prior to spreading over coated and blocked coverslips.

For phalloidin labelling of the actin cytoskeleton, platelets were permeabilised using 0.1% (w/w) Triton X-100 for 5 min after fixation, washed × 3 with PBS before labelling with 200 U/mL Alexa Fluor-488 conjugated phalloidin for 30 min.

### DIC imaging

Platelets were imaged by Köhler illuminated Nomarski differential interference contrast (DIC) optics using a Nikon eclipse Ti2 inverted microscope, equipped with a Nikon DS-Qi2 camera, and visualised using a 100 × oil immersion objective lens. NIS Elements software was used for image capture. All training, test and inhibitor images are available via The University of Reading Research Data Archive repository: https://doi.org/10.17864/1947.000332.

### Automated segmentation

Platelets were segmented in each of the DIC images using a modified version of the Usiigaci pipeline^20^. A training data set of 120 DIC images was manually curated using ImageJ^21^. The original 16-bit DIC images with dimensions 2424 × 2424 were rescaled and converted to 970 × 970 8-bit images to reduce the file size. The LOCI plug-in for ImageJ was then used to label the individual platelets within each of the DIC images. In line with manual analysis, touching platelets were excluded from the manual annotations to avoid boundary ambiguity. The same process was repeated for a further 12 images to form an independent test set. Finally, the hyperparameters and learning rate structure were kept constant for all the models as described in Butler et al.^22^, and included a filter whereby objects containing an area of < 250 pixels, or any pixels within a 10 pixel range of the image edge, were eliminated from the analysis. A quality control step allows assessment of automated segmentation prior to data output. All code and corresponding data are available at: https://github.com/george-butler/Automated_DIC_platelet_analysis. All graphs and statistical tests were performed using Prism software version 8 for Windows (GraphPad Software, San Diego, California USA, www.graphpad.com).

### Fluorescent image analysis

Phalloidin labelled platelets were imaged using a Nikon eclipse Ti2 inverted microscope using a green fluorescent protein (GFP) filter (Excitation wavelength 450–490 nm). NIS Elements software was used for both image capture and fluorescent analysis (NIS-Elements AR Analysis, version 5.21.02). Briefly, each image was converted into a binary image whereby global thresholds were set. Touching platelets were excluded by setting a size criterion, and fluorescent staining irregularities were corrected by selecting the fill criteria.

### Manual annotations

All manual annotation was performed blinded. The perimeter of all platelets in each image were manually annotated using a pen tablet (Wacom Intuos). All manual annotators were provided with the same protocol, a virtual demonstration and requested to practise on a subset of images prior to commencing annotations. All manual annotators were instructed to avoid annotating touching platelets.

## Results

### A practical increase in training data size will yield a significant increase in network performance

**Figure 1:**
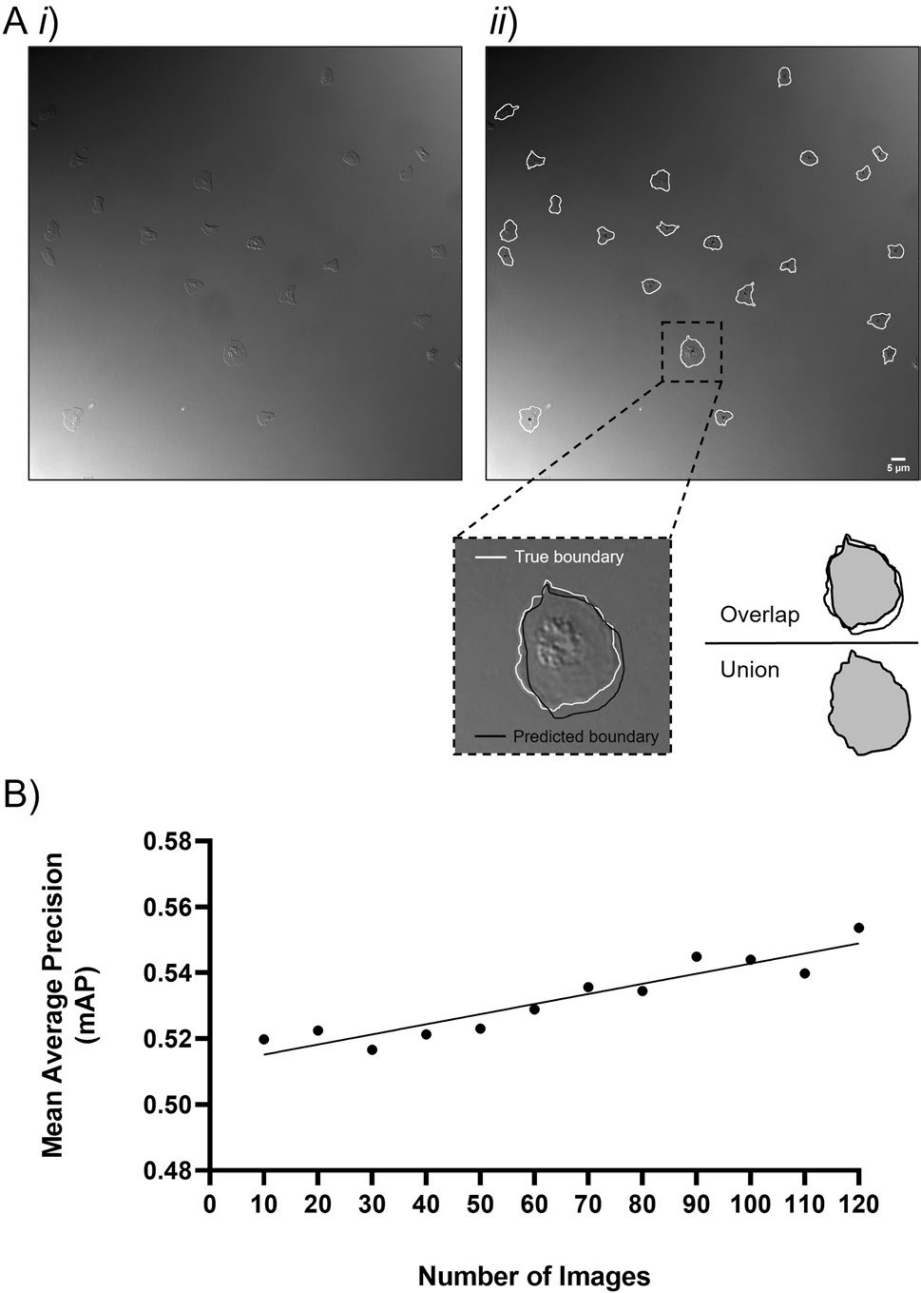
Increasing the number of images in the training set increases the performance of the CNN. Training consisted of a supervised approach to assist the training of the CNN. Representative DIC image (**Ai**). Representative manually annotated image (**Aii**). The true platelet boundary (white) as identified by manual annotations was compared to the predicted boundary (black) of the CNN (**Aii**). Comparing these boundaries evaluated the extent of overlap and returned mean average precision (mAP) whereby increasing increments of 10 images assessed the network performance up to 120 images (**B**). A linear regression model shows mAP increased with an increasing training set. Data represents the mean of three independently trained models.

Firstly, the mean average precision (mAP) was used to evaluate the effect of increased training data on the network performance. The mAP compares the predicted platelet boundary, as outputted by the CNN, against the true platelet boundary as identified by the manual annotations (Fig. 1Ai–ii). The training data size was then increased from 10 to 120 images with a 10-image increase at each interval. The mAP at each interval was then calculated as the average across 3 independently trained models (Fig. 1B). As expected, increasing the amount of training data monotonically increases the network performance. That is, the accuracy of the predicted platelet boundary, identified by the trained CNN, increased as the number of training images increases.

A mAP output of ≥ 0.5 is predictive of a sufficiently good model performance^23,24^, and with the experimental setup presented here, a strong performance (0.55 ± 0.01) was achieved with a training set of 120 images (Fig. 1B). Although this data does not indicate a saturation point for maximal performance, the size of this training set was realistic and manageable to curate. Furthermore, higher order polynomial models did not describe the data any better when compared to a simple linear regression model (Supplementary Fig. S1). This therefore suggests that the saturation point was not imminent and that considerably more training data would be needed to reach the maximal segmentation performance. As a result, the 3 models trained with 120 training images were used in an ensemble approach for all future segmentation^18^.

### Trained CNN removes variation in manual annotations

**Figure 2:**
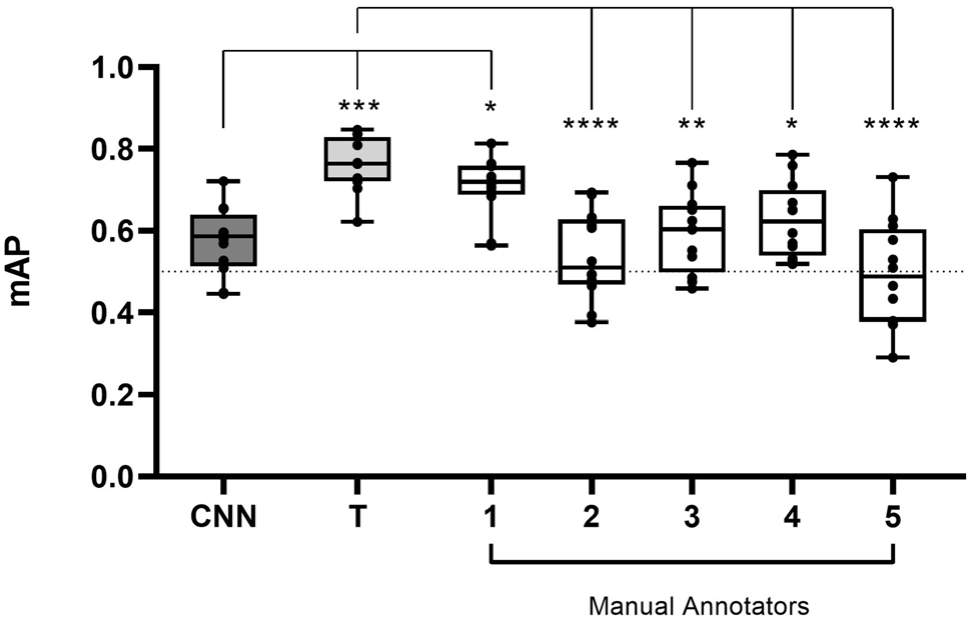
The CNN removes large variation between manual annotators. Six independent annotators, including manual annotators (1–5) and a network trainer (T), manually outlined the perimeter of platelets in 12 images. The mAP of the CNN was compared to all manual annotators. The mAP of the trainer was compared to manual annotators 1–5. The mean ± SD of the 12 images was plotted and analysed using one-way ANOVA with Bonferroni post-test. *p ≤ 0.05, **p ≤ 0.01, ***p ≤ 0.001, ****p ≤ 0.0001.

To test the performance of the CNN when compared to multiple manual annotators the outputs of six independent platelet annotators were assessed (Fig. 2); whereby ‘T’ represents a network trainer, and 1–5 represent additional annotators. All annotators, including the trainer, were presented with the same 12 images, and instructed to manually annotate the perimeter of individual platelets in each image. The mAP of the CNN was compared to the mAP of the trainer and each manual annotator. The higher the mAP score, the more accurate the CNN or annotator was when detecting platelets in each image.

Unsurprisingly, the mAP of the trainer was generally higher than that of other manual annotators—that is to say, the CNN’s output was similar to that of the person whose annotations were used to train the CNN. Despite the mAP of the trainer being significantly different to annotators 2–5, the mAP of these annotators (0.49 ± 0.13—0.63 ± 0.09) were similar and consistent with the mAP of the CNN (0.57 ± 0.08). There was also no significant difference in mAP between the trainer and annotator 1, indicating that the mAP was similar between these individuals. Overall, the high degree of variation between the manual annotators supports the need for an automated CNN to ensure data outputs are non-biased and reproducible across experiments.

**Figure 3:**
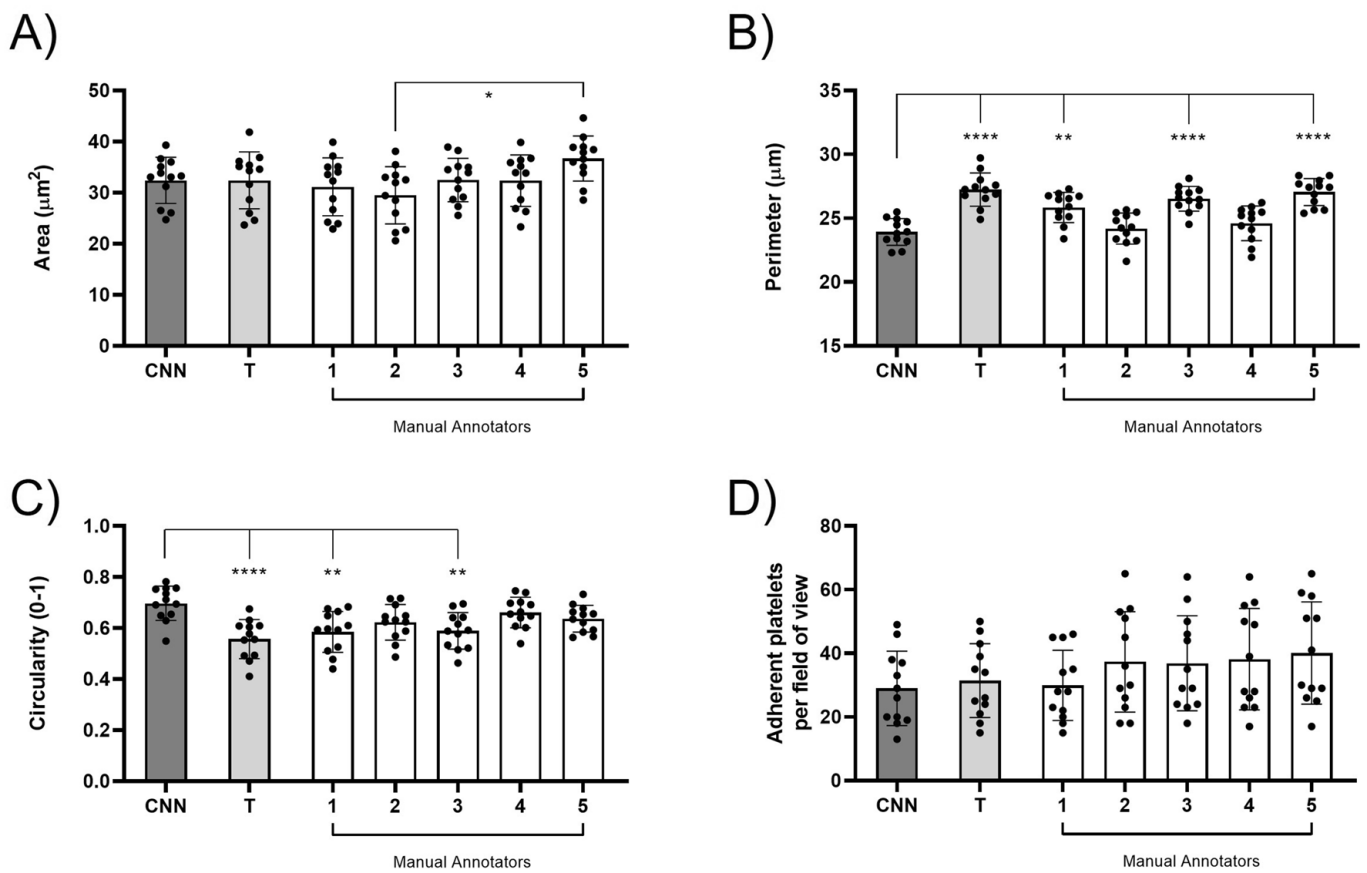
The CNN identifies differences in some metrics of morphology. Commonly evaluated platelet metrics were exported to determine platelet surface area (**A**), platelet perimeter (**B**) platelet circularity (**C**) and adhesion (**D**) in 12 images. The automated output of the CNN was compared to a trainer (T) and manual annotators (1–5). The mean ± SD of the 12 images was plotted and analysed using one-way ANOVA with Bonferroni post-test. *p ≤ 0.05, **p ≤ 0.01, ***p ≤ 0.001, ****p ≤ 0.0001.

Spread area, perimeter and circularity are commonly assessed parameters of platelet spreading assays. When these parameters were compared between the trainer, annotators and the CNN, the spread area of platelets was found to be consistent, yet significant differences were observed when assessing platelet perimeter and circularity when spread over fibrinogen (Fig. 3A–C). The CNN successfully detects the spread area of platelets to be 32.4 ± 4.5 μm^2^ (Fig. 3A). This corresponds with the spread area for platelets interacting with fibrinogen reported in the literature^25^. When the outputs from the manual annotators are compared, annotator 5 appears to estimate a larger spread area (36.7 ± 4.4 μm^2^) when compared to the spread area estimated by annotator 2 (29.5 ± 5.6 μm^2^). The mAP of the spread area is overall consistent, yet an automated CNN would avoid biased and inconsistent analyses.

However, when the perimeter was analysed, the CNN appears to lose detail from the platelet perimeter. This is reflected by the reduced perimeter when compared to the trainer and annotators 1, 3 and 5 (Fig. 3B). The platelet perimeter determined by the CNN is 23.9 ± 1.0 μm, and when compared to the manual annotators, there is a significant increase in perimeter measurements identified by the trainer and manual annotators 1, 3 and 5 (25.8 ± 1.2 μm–27.3 ± 1.3 μm). This suggests that the CNN has difficulty in detecting the intricate details of the platelet perimeter which the human eye can observationally identify. This variability in platelet perimeter in turn affects the data outputs for circularity (Fig. 3C). Circularity is a normalised ratio between the area and perimeter^26^. Circularity can represent changes in platelet morphology with filopodia containing platelets having a lower circularity score when compared to fully spread platelets which have a high circularity score. The circularity for each platelet was calculated with a measurement between 0 and 1, where 0 is not circular and 1 is a perfect circle. An increase in circularity value was observed for the CNN (0.7 ± 0.07) when compared to the trainer and manual annotators 1 and 3 (0.56 ± 0.08–0.59 ± 0.07). This highlights that, since some finer detail in platelet perimeter is missed by the CNN, circularity score is directly impacted. No difference in cell adhesion was observed between the CNN and the manual annotators (Fig. 3D).

In summary, the CNN can accurately identify platelets and can determine the spread platelet area. However, differences are observed for perimeter and circularity, indicating that automated outputs should be carefully interpreted and validated.

### Trained CNN detects extremes in platelet morphology

We next sought to identify if the CNN could detect extremes in platelet cell shape. Platelet morphologies are often investigated in spreading experiments since they can be suggestive of platelet abnormalities or disorders.

**Figure 4:**
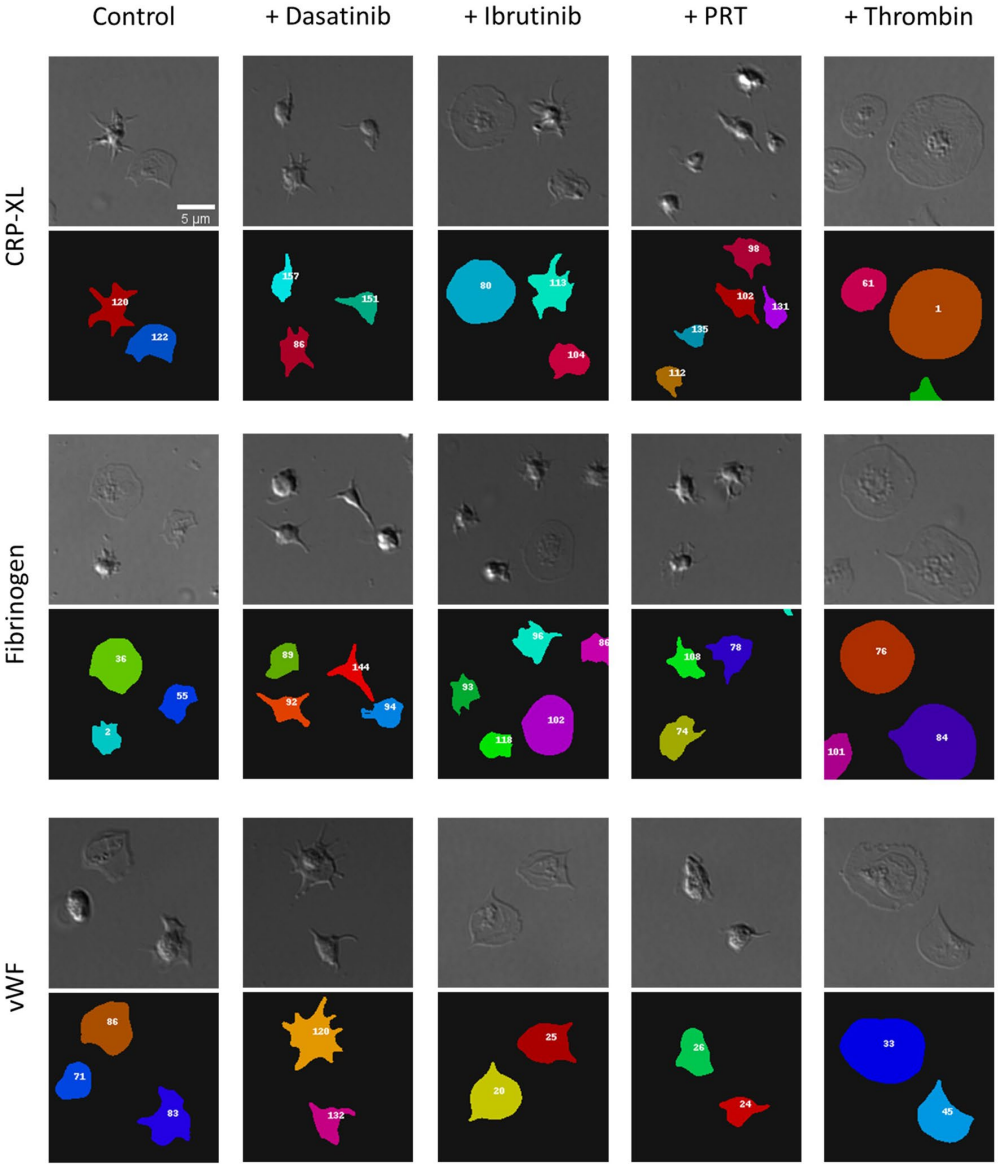
CNN can successfully identify inhibitor and agonist-induced changes in platelet morphology. Washed platelets (1 × 10^7^/mL) were spread over three different substrates (CRP-XL, fibrinogen or vWF) in the presence of either Dasatinib [10 μM], Ibrutinib [1 μM], or PRT-060318 [5 μM] inhibitor, thrombin [0.1 U/mL], or in the absence of inhibitor or agonist (control) to assess if the CNN could measure extremes in cell morphology. Representative cropped DIC images show the extremes in platelet cell shape, whilst corresponding segmented images demonstrate the cell morphology identified by the CNN. Scale bar represents 5 μm.

To do this, washed platelets were spread over three different substrates consisting of the synthetic peptide CRP-XL, and glycoproteins fibrinogen or vWF (Fig. 4). Washed platelets were also treated with a selection of inhibitors known to impair platelet spreading; ibrutinib^27^, dasatinib^28,29^ or PRT-060318^30^ which inhibit, Btk, Src family kinases and Syk respectively. In contrast, thrombin, a potent platelet agonist which induces platelet activation independently of the adhesion receptors that control platelet spreading, was used to induce fully spread platelets by activating protease-activated receptors (PARs)^30–32^. Representative images indicate these extremes in platelet morphology, and the corresponding segmented images detail the morphology as identified by the CNN (Fig. 4).

**Figure 5:**
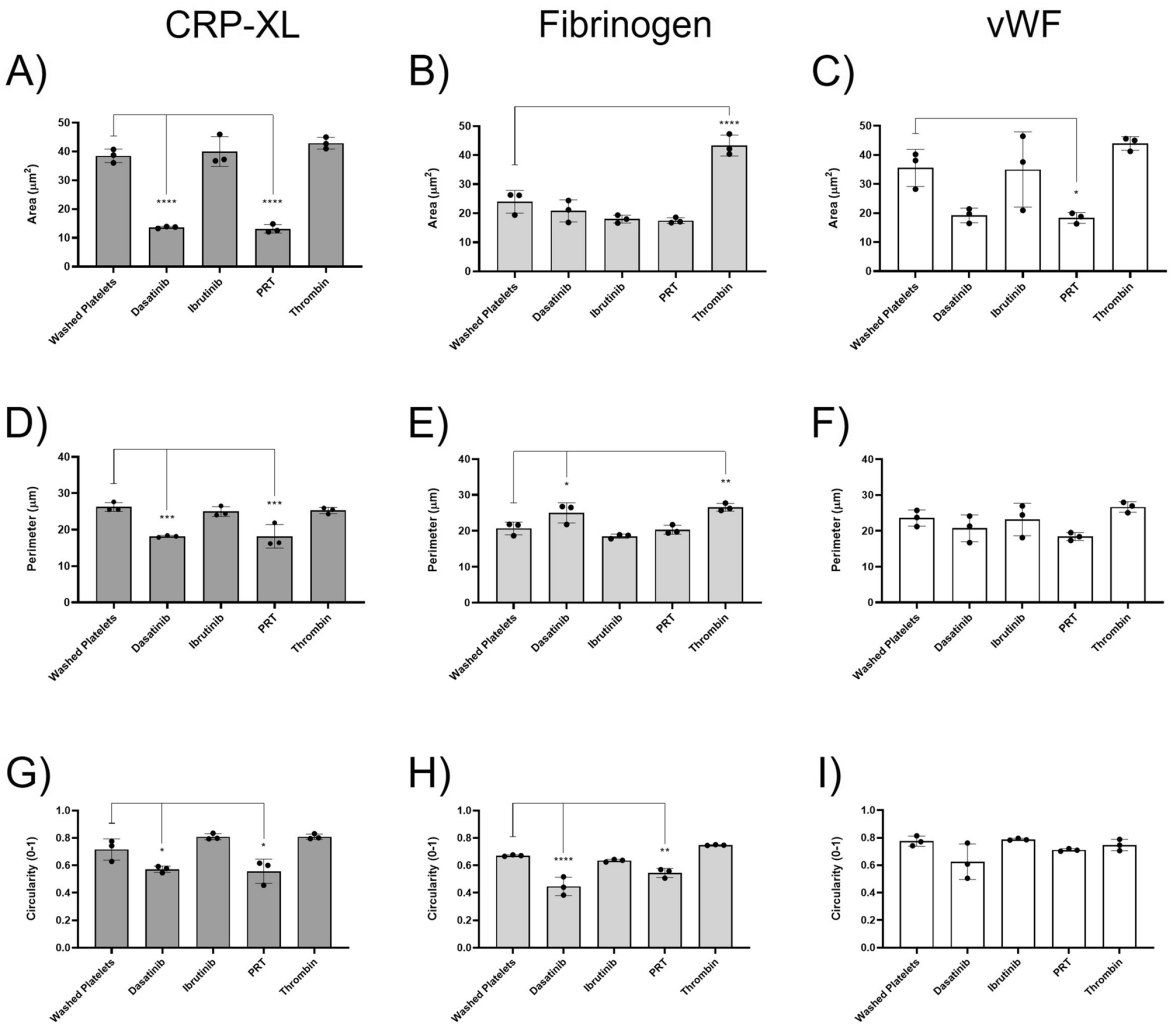
CNN can successfully quantify inhibitor and agonist-induced changes in platelet morphology. Platelet spread area (**A**–**C**), platelet perimeter (**D**–**F**) and platelet circularity (**G**–**I**) were quantified over the three different substrates (CRP-XL (**A**, **D**, **G**), Fibrinogen (**B**, **E**, **H**) and vWF (**C**, **F**, **I**)). The mean ± SD of three experimental replicates (n = 3), whereby each experimental replicate was the mean of five fields of view, were plotted and analysed using one-way ANOVA with Bonferroni post-test. *p ≤ 0.05, **p ≤ 0.01, ***p ≤ 0.001, ****p ≤ 0.0001.

The data outputs from the CNN are consistent with non-automated approaches reported in the literature^27,30,33–37^. The CNN identified that dasatinib (13.66 ± 0.34 μm^2^) and PRT-060318 (13.16 ± 1.48 μm^2^) resulted in a significant decrease in platelet spread area when compared to control platelets spread on CRP-XL (38.52 ± 2.34 μm^2^) (Fig. 5A). Dasatinib is an inhibitor of Src family kinases (SFKs) known to impair collagen-induced signalling^33,34^, whilst PRT-060318 is a Syk inhibitor previously shown to reduce platelet spreading over collagen^30,35^. Ibrutinib had no inhibitory effect on platelet spreading on CRP-XL (Fig. 5A), which is supported by studies which suggest that the kinase activity of Btk does not play a major role downstream of GPVI^27,33^. No further increase in spread area was found in the presence of thrombin, suggesting that control platelets were fully spread.

When platelets were spread on fibrinogen the CNN identified that thrombin (43.31 ± 3.60 μm^2^) resulted in a significant increase in platelet spread area when compared to control platelets (23.97 ± 3.94 μm^2^) (Fig. 5B). This is a result of thrombin initiated integrin inside-out signalling, leading to enhanced activation and binding of the integrin αIIbβ3 to fibrinogen and increased platelet spreading^36^. Dasatinib, ibrutinib and PRT-060318 had no inhibitory effect on the platelet spread area over fibrinogen. PRT-060318 was found to have a significant inhibitory effect on the platelet spread area over a vWF substrate (18.38 ± 1.85 μm^2^) when compared to control platelets (35.55 ± 6.38 μm^2^) (Fig. 5C). The role of Syk down stream of glycoprotein Ib (GPIb) is controversial but Syk deficient platelets display inhibited platelet spreading on vWF^37^. Dasatinib, ibrutinib and thrombin had no significant effect on platelet spreading over vWF (Fig. 5C).

When observing perimeter measurements (Fig. 5D–F), the CNN identified that dasatinib (18.19 ± 0.25 μm) and PRT-060318 (18.18 ± 3.18 μm) resulted in a significant decrease in platelet perimeter when compared to control platelets on CRP-XL (Fig. 5D). Further supporting the inhibition of Src and Syk signalling pathways which are known to impair platelet spreading on collagen. There was no difference in perimeter measurements between platelets treated with ibrutinib or thrombin.

The CNN identified that platelets treated with dasatinib (24.99 ± 2.82 μm) have a significantly increased perimeter when compared to control platelets (20.68 ± 1.73 μm) on fibrinogen (Fig. 5E). Consistent with the literature, dasatinib results in small spikey platelets, described here by a decreased spread area and an increased perimeter. Additionally, when compared to control platelets, there are significant increases in the perimeter when treated with thrombin (26.52 ± 1.07 μm), which is consistent with an increase spread area due to activated αIIbβ3 (Fig. 5E). No inhibitory effects on the perimeter were observed in the presence of Ibrutinib or PRT-060318 on fibrinogen, while the CNN identifies no difference in platelet perimeter when spread on vWF (Fig. 5F).

Circularity scores were also outputted by the CNN, which identified that dasatinib (0.57 ± 0.02) and PRT-060318 (0.56 ± 0.09) were significantly decreased and less circular than control platelets (0.72 ± 0.08) on CRP-XL (Fig. 5G), consistent with reports that these inhibitors result in small and non-circular platelets as a result of abolished lamellipodia formation. As with spread area and perimeter, there were no inhibitory effects in the presence of ibrutinib and thrombin, suggesting that platelet spreading was not impacted by either. The CNN also identifies that circularity for both dasatinib (0.45 ± 0.07) and PRT-060318 (0.55 ± 0.03) are significantly decreased when compared to control platelets (0.67 ± 0.01) on fibrinogen (Fig. 5H). There was no inhibitory effect by ibrutinib or reactivity to thrombin on fibrinogen, suggesting that ibrutinib had no effect on platelet circularity, and that platelets were fully spread in the presence of thrombin. Similar to spread area and perimeter, no significant differences were observed between control, inhibited platelets and activated platelets on vWF (Fig. 5I).

To determine if the CNN could be used to quantify mouse platelet morphology, mouse platelets were spread over fibrinogen. As with human platelets, the CNN was able to segment mouse platelets without further training (Supplemental Fig. S2).

These data demonstrate that the trained CNN successfully detects extremes in platelet morphologies over different substrates when pre-treated with inhibitors known to impair platelet spreading.

### CNN removes bias observed between manual annotators when evaluating inhibitor and agonist-induced changes in platelet morphology

The five manual annotators annotated platelets spread over fibrinogen in the presence or absence of inhibitor or agonist. All manual annotators were blinded to the image selection and presented with identical sets of images. Fibrinogen was chosen as a substrate which showed subtle phenotypic changes in platelet morphology in the presence of inhibitors when compared to control platelets, with the aim to expose genuine similarities or differences between the five manual annotators.

**Figure 6:**
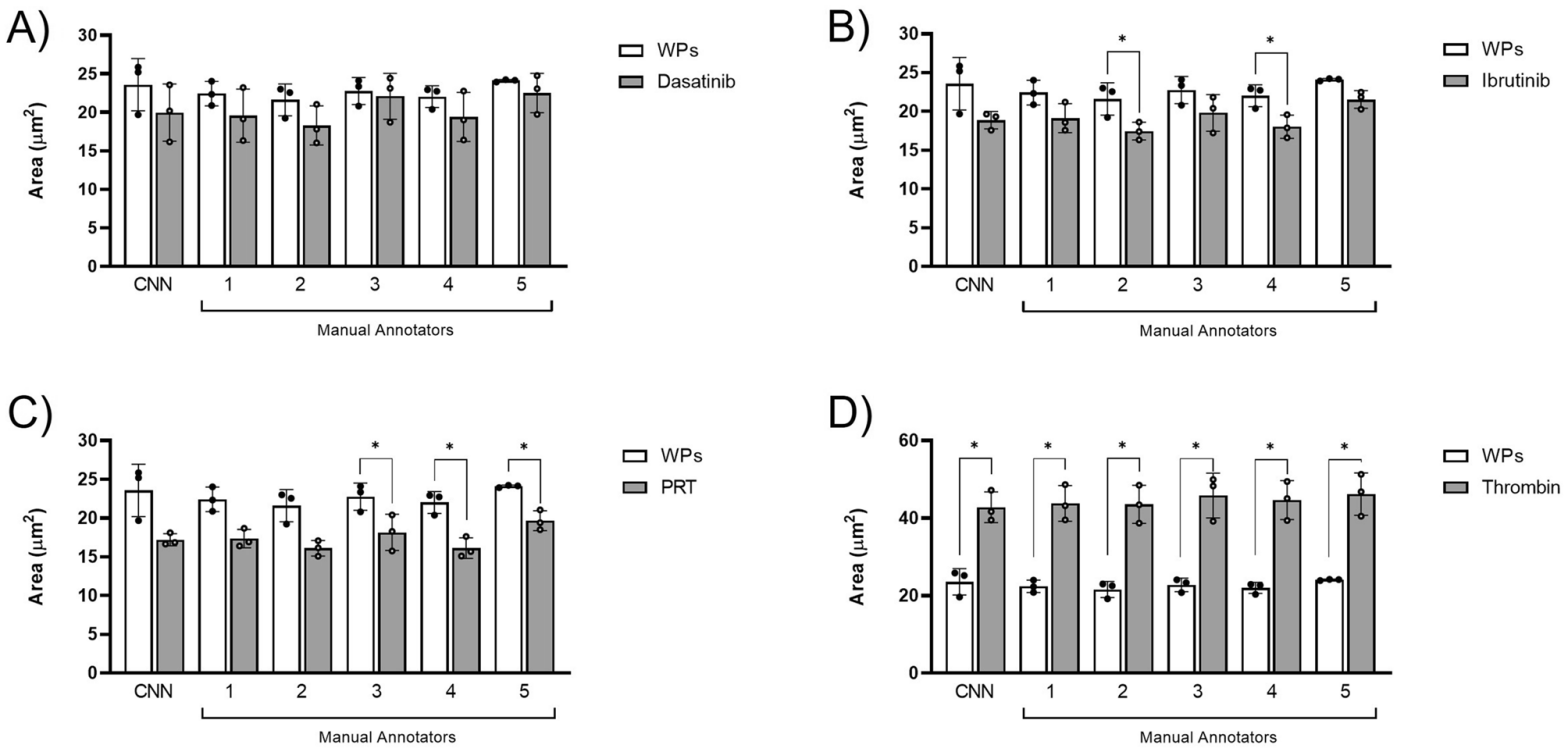
CNN avoids the bias observed between different manual annotators when evaluating inhibitor and agonist-induced changes in platelet morphology. Control washed platelets (WPs) quantified by the CNN and manual annotators were directly compared to platelets treated with dasatinib (**A**), ibrutinib (**B**), PRT-060318 (**C**) and thrombin (**D**). The mean ± SD of three experimental replicates (n = 3), whereby each experimental replicate was the mean of three fields of view, were analysed using a paired two-tailed T-test. *p ≤ 0.05, **p ≤ 0.01, ***p ≤ 0.001, ****p ≤ 0.0001.

The control data outputs from the CNN and the manual annotators for washed platelets were directly compared to either dasatinib (Fig. 6A), ibrutinib (Fig. 6B), PRT (Fig. 6C) or thrombin treated platelets (Fig. 6D). There were no significant differences in surface area when directly comparing washed platelets against dasatinib treated platelets for both the CNN and all manual annotators (Fig. 6A). Suggesting that firstly, there were little changes in surface area between these two treatments, but secondly, that neither CNN nor manual annotators interpreted spread platelet area differently.

However, when comparing washed platelets to ibrutinib treated platelets (Fig. 6B), there are no significant differences observed for the CNN, and manual annotators 1, 3 and 5. However, there were significant differences when comparing washed platelets to ibrutinib treated platelets for manual annotators 2 and 4, suggesting that these annotators interpreted the images differently to the CNN and the other annotators (Supplemental Fig. S3).

Differences were also observed when comparing washed platelets to PRT treated platelets (Fig. 6C). No significant differences were observed for the CNN, and manual annotators 1 and 2. However, there were significant differences when comparing washed platelets to PRT treated platelets for manual annotators 3, 4 and 5. Again, suggesting that these manual annotators interpreted the changes to platelet morphology differently than the CNN and other annotators (Supplemental Fig. S3).

Conversely, when comparing washed platelets to thrombin treated platelets (Fig. 6D), there were significant increases in surface area for the CNN and all manual annotators. This suggests that the CNN and all manual annotators interpreted the spread area of platelets treated with thrombin similarly.

Together, this identifies that there are significant differences in the scientific conclusions drawn between manual annotators for the analysis of platelet spreading in the presence of ibrutinib and PRT. This variation may be due to different interpretations of platelet morphology by the annotators or by an increased risk of Type II error due to the small sample size. However, overall, this data provides evidence that image analysis by the CNN, which is comparable to some manual annotators in this study, can remove potential bias associated with time consuming manual analyses.

### Comparison of CNN outputs with fluorescently labelled platelets

**Figure 7:**
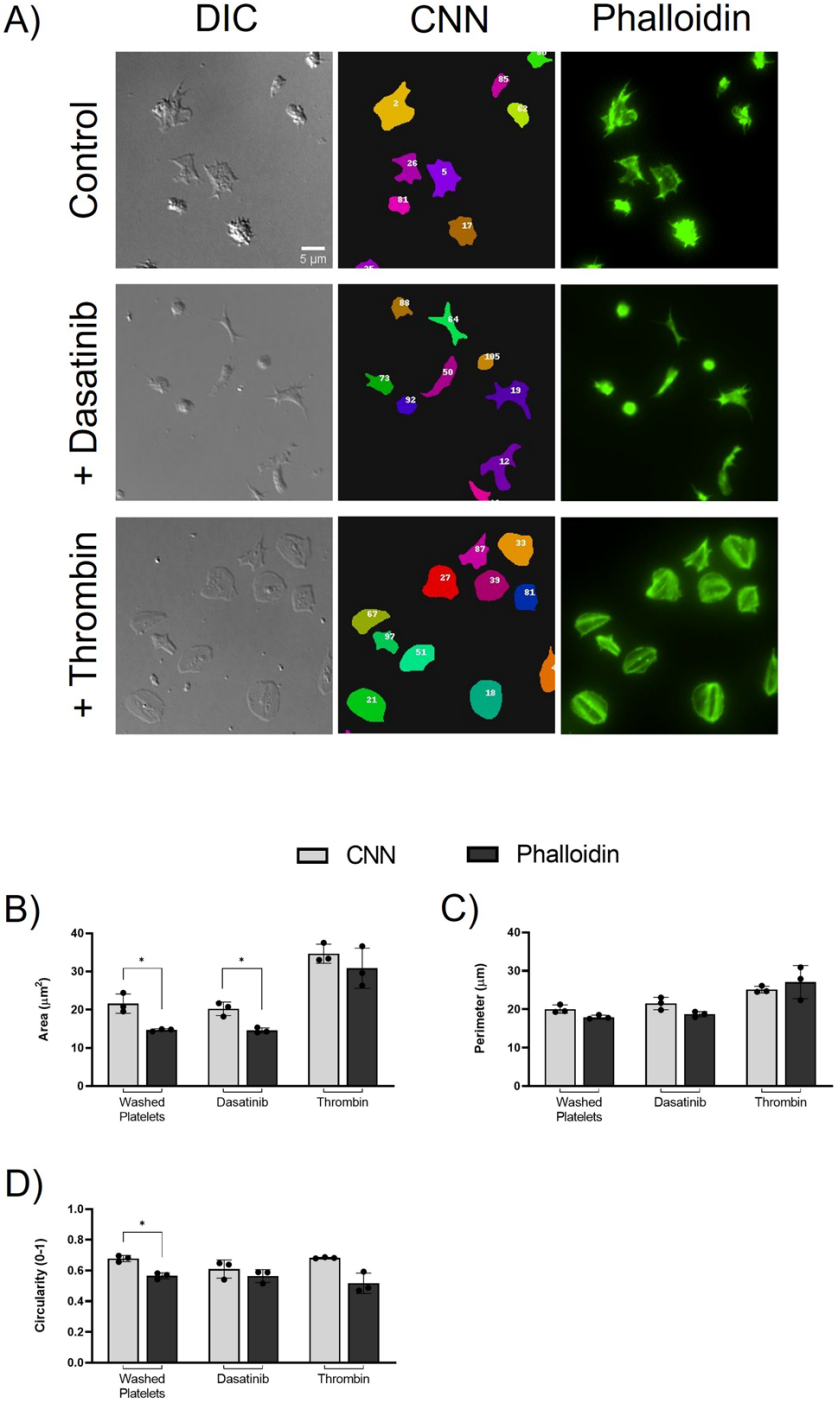
Comparison of platelet morphology by the CNN to fluorescently labelled platelets. Washed platelets, treated with and without dasatinib [10 μM], and thrombin [0.1 U/mL] were spread over a fibrinogen substrate. Representative images (**A**) show a DIC image, matched CNN segmentation, and matched fluorescently labelled image of platelets stained with Alexa-Fluor 488 conjugated phalloidin [0.3 U/mL]. CNN data was compared to fluorescent analysis for spread platelet area (**B**), perimeter (**C**) and circularity (**D**). The mean ± SD of three experimental replicates (n = 3), whereby each experimental replicate was the mean of three fields of view, were analysed using paired two-tailed T-tests. *p ≤ 0.05, **p ≤ 0.01, ***p ≤ 0.001, ****p ≤ 0.0001.

We next sought to compare the CNN output to the quantification of platelet morphology by using platelets fixed, permeabilised and labelled with Alexa-Fluor 488 conjugated phalloidin. No difference is observed in CNN quantification between fixed platelets, and fixed and permeabilised platelets (Supplemental Fig. S4). Representative images show the DIC image together with the segmentation of platelets as identified by the CNN, and fluorescently labelled permeabilised platelets by phalloidin labelling (Fig. 7A).

While the scientific conclusion of the CNN and phalloidin quantification outputs were the same, a significant decrease in platelet spread area for washed platelets and dasatinib treated platelets was seen in phalloidin labelled platelets when compared to the CNN segmentation (Fig. 7B). This may, in part, be due to the nonuniform staining of the actin filaments in spreading platelets and the need to threshold the fluorescent images. There was no difference in the spread area of platelets as measured by the CNN or phalloidin labelling in the presence of thrombin.

No differences were observed when comparing platelet perimeter quantified by the CNN to phalloidin labelling of washed platelets in the presence or absence of dasatinib or thrombin (Fig. 7C). However, there was a significant decrease in platelet circularity when comparing the CNN output with phalloidin labelled washed platelets (Fig. 7D). As shown in Fig. 3, discrepancies with circularity quantified by the CNN have been identified, so it is unclear if this is due to the smoothing effect of the CNN. No significant difference was seen between the CNN and phalloidin labelling when calculating the circularity in platelets treated with dasatinib or thrombin.

This data demonstrates that while outputs from the CNN analysis cannot be directly compared to phalloidin analysis, the scientific conclusion using each method is the same.

## Discussion

Here we demonstrate, for the first time, a method for the fully automated platelet morphology analysis of DIC images by the implementation of a CNN. In this study we demonstrate that (1) a manageable increase in training material will improve CNN performance when assessing mAP; (2) the CNN is consistent in quantifying the spread area of platelets when directly compared to manual annotators; (3) the automated CNN has potential limitations in that its measurements of other commonly used platelet metrics, perimeter and circularity, are not always comparable to those of manual annotators; and (4) the CNN is capable of segmenting and quantifying extremes in platelet morphologies when inducing inhibition or activation of biologically important pathways known to impact platelet spreading. Further, we identify that the quantification of platelet spreading morphology by different manual annotators can result in different scientific conclusions being drawn. The CNN therefore removes the potential bias associated with manual annotations.

This work presents a fully automated platelet spreading analysis approach facilitated by a supervised training set consisting of 120 DIC images. Although the curation of the initial training material can be time consuming, the substantial increase in the mapping function of the CNN when increasing the training material is evident. However, training material is typically generated by one or two expert annotators which can potentially incur bias of both the training material selected and manual interpretations. A community effort is recommended to minimise bias associated with training material^38^. Nonetheless, this raises the question of how many training images are required to reasonably train a CNN. While this question has not been answered in full, we have demonstrated that a realistic size of training images returned a strong performance as assessed by mAP, and successfully identified platelets during multiple independent experiments. This suggests that the CNN can easily be generalised to different experimental set ups.

In addition to being generalisable, this computational model removes user variability and bias associated with subjective decisions by manual annotators^38^. We found significant subjective variability between individuals during manual segmentation of DIC images, whereby the shadow artefact used to enhance contrast may contribute to a difference in image interpretation. Similar to its mAP performance, the CNN successfully measured platelet spread area, a commonly used metric to assess platelet function and the impact of anti-platelet therapies. Spread area was consistent with manual annotators, and automated outputs were directly comparable with published data. Therefore, this CNN presents a practical, fast and robust method to automate large-scale platelet spreading analyses.

However, other commonly extracted measurements, such as platelet perimeter and circularity were not directly comparable to those obtained by manual segmentation. Quantitative outputs from the CNN identify a decreased perimeter, and consequently an increased circularity when compared to a trainer and 5 independent annotators. This may be a direct limitation of the pseudo shadow effect of the DIC imaging technique as discussed previously. This shadow artefact could be impacting on feature extraction by creating a smoothing effect which impacts segmentation, causing a difference between manual and automated measurements, and hence identifying limitations of a CNN.

Nonetheless, the CNN performs extremely well when quantifying extremes in platelet morphology when inducing inhibition to Syk and Src activation pathways, and by thrombin induced platelet spreading. The CNN successfully detected morphological extremes which may be typical of platelet defect phenotypes, which are abundantly researched in the platelet field. Additionally, automated analysis modalities such as this, may aid the clinical stratification of individuals at risk of bleeding or thrombosis whereby platelet spreading analyses are currently missing from standard haematology approaches due to the complexity of analysis and experience required of the researcher.

Overall, the application of deep learning models for biological image analysis has become increasingly prevalent in recent years and, as demonstrated in this study, abrogates time consuming manual analyses and removes individual subjectivity and bias. Yet, in an era whereby many science domains are producing unprecedented amounts of data, we highlight some challenges and limitations which should be considered prior to using automated outputs. Firstly, one of the challenges which remains in computational models is how to manage differences between data sets e.g., differing contrast and focus. Noise within images at acquisition can directly impact the learning accuracy and output accuracy of a CNN^39,40^. Secondly, CNNs require a large amount of generalised training material which can be time consuming to curate yet, as demonstrated here, a realistic increase in training material substantially increases model performance^16^. Thirdly, all datasets will contain a level of bias. For any image, only a limited number of scenarios will occur. A CNN, therefore, may be biased towards a particular training set^41^. Although, data augmentation can assist training by artificially inflating the original training material, some studies demonstrate that performance is not improved when compared to those trained using real images^42^. These points highlight how fundamental generalised training material is to the performance of a CNN.

The CNN has the potential to be adapted to alternative imaging modalities, such as phase contrast^43^, if new training material is generated. Furthermore, the successful segmentation of adhered platelets by deep learning could open up the potential to exploit live cell tracking of dynamic platelet processes which are involved in platelet activation and thrombus formation. A limiting factor in live cell tracking by deep learning, and which adds additional computational complexity, is that most cell types will undergo cell division. However, platelets are non-dividing cells, and recent studies have shown successful live cell tracking in other non-dividing cell lines^44,45^. Interestingly, an active area of research has also involved using CNNs to investigate cell–cell interactions^46–48^, and although a quality control step has been maintained in the approach presented here to allow users to quickly assess and remove instances of platelet-platelet interactions, this may be an area of interest in the future.

In summary, the implementation of a CNN to enable automated analysis of platelet morphology removes subjective bias, which is associated with time consuming manual analysis methods, and offers the potential to deliver substantial increases in the quantity and consistency of large platelet data sets, where throughput has previously been limited.

Caution should be employed to fully understand the possible drawbacks of CNNs, and to carefully validate automated outputs. Nevertheless, an automated CNN is advantageous, and given the ease to make adaptations to the CNN, we present the potential for robust and collaboratively distributed platelet analyses between laboratories.

## Supplementary Information

Supplementary Information.
