# Supplementary material for: Fully automated platelet differential interference contrast image analysis via deep learning
Source: Sci Rep. 2022 Mar 17;12:4614. doi: 10.1038/s41598-022-08613-2 (PMC8931011; doi:10.1038/s41598-022-08613-2)
Supplement: Supplementary file 1 — Supplementary Information. [file 41598_2022_8613_MOESM1_ESM.docx]

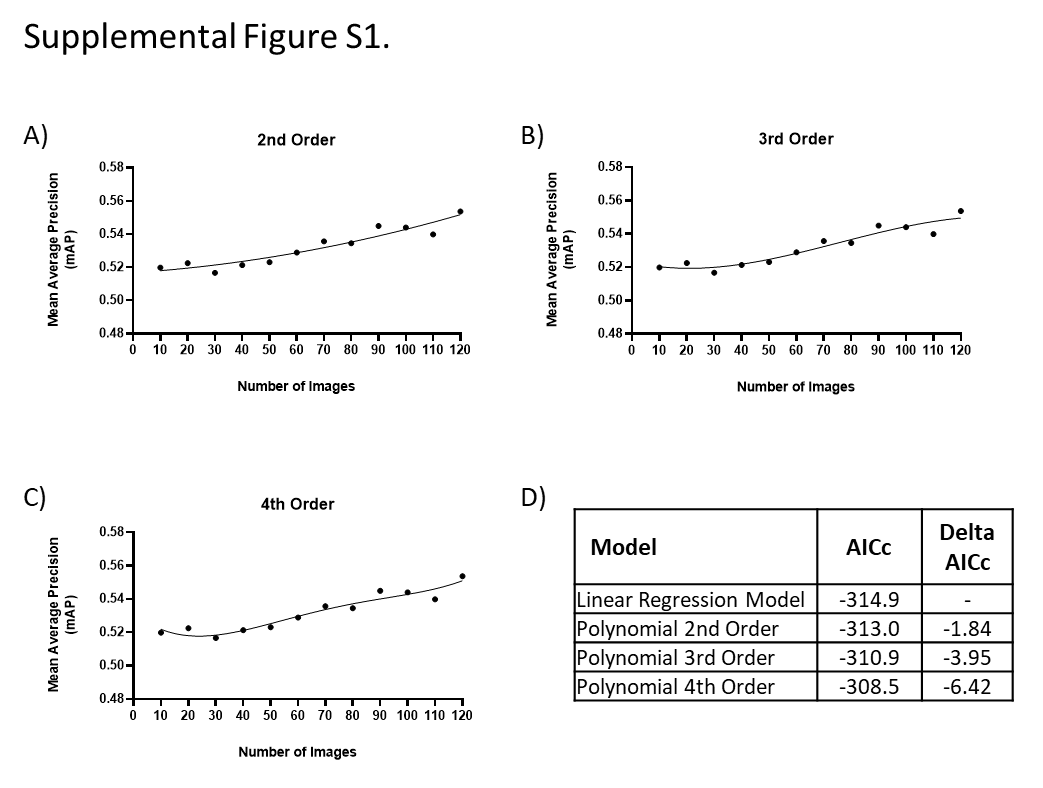


**Supplemental Figure S1. Polynomial models are not significantly different to a linear regression model.**

Polynomial regression models of the 2nd order (A), 3rd order (B), and 4th order (C) are not significantly different to the linear regression model (Figure 1). Indicating the relationship between independent and dependent variables is no further defined; exemplifying no reason to reject the linear regression model. A corrected Akaike information criterion value (AICc) identifies a smaller value for the linear regression model, which is representative of a better model fit (D). The delta AICc highlights the increasing difference in model fit when the polynomial models are compared to the linear regression model. Data shown as the mean of three independently trained models.


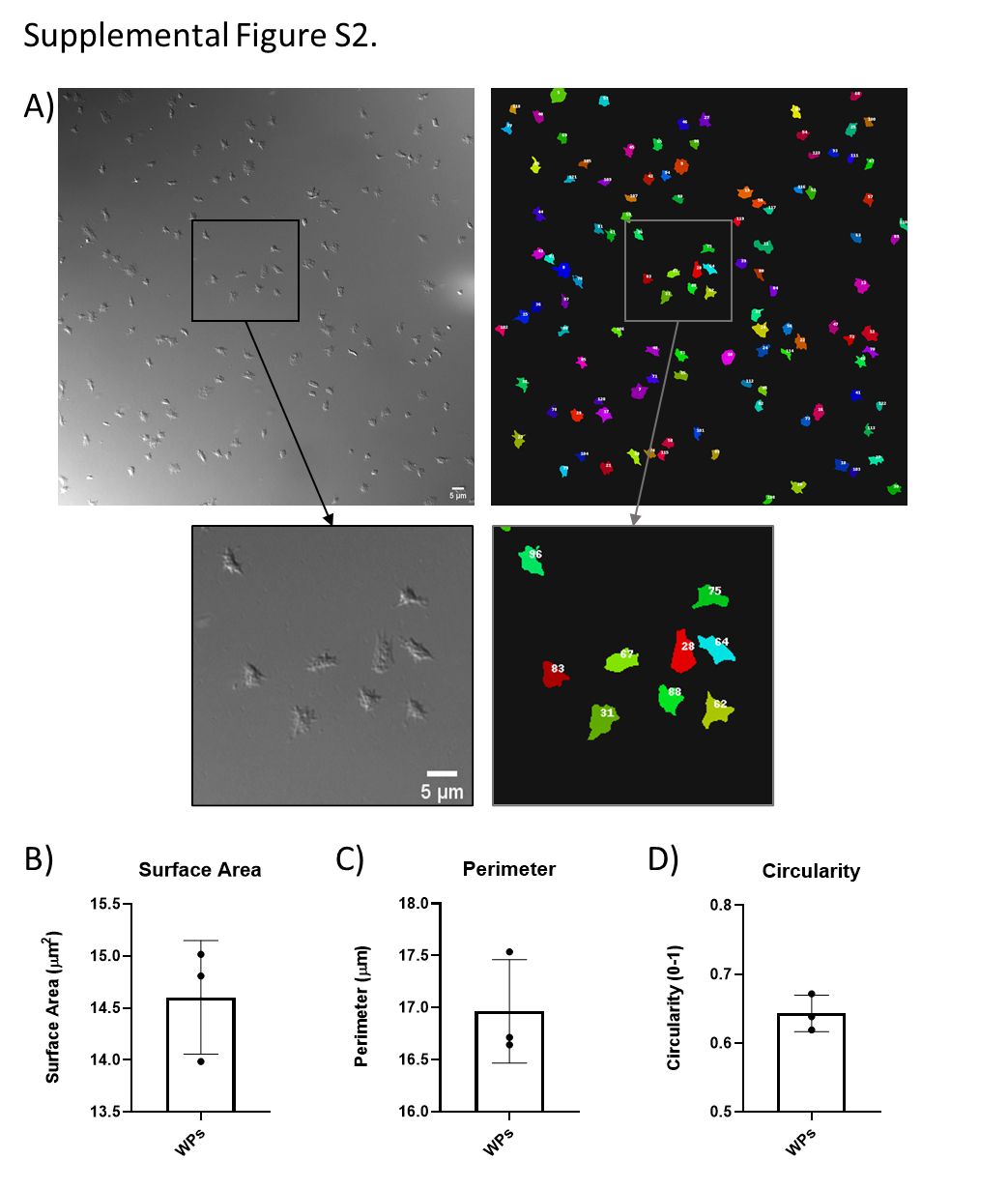


**Supplemental Figure S2. CNN successfully quantifies mouse platelet morphology.**

Washed mouse platelets (1 x 10^7^ /mL) were spread over fibrinogen to assess CNN segmentation and quantification of mouse platelets. A representative DIC image provides an example of mouse platelet spreading with a matched segmented prediction by the CNN (A). The CNN quantified surface area (B), perimeter (C) and circularity (D).


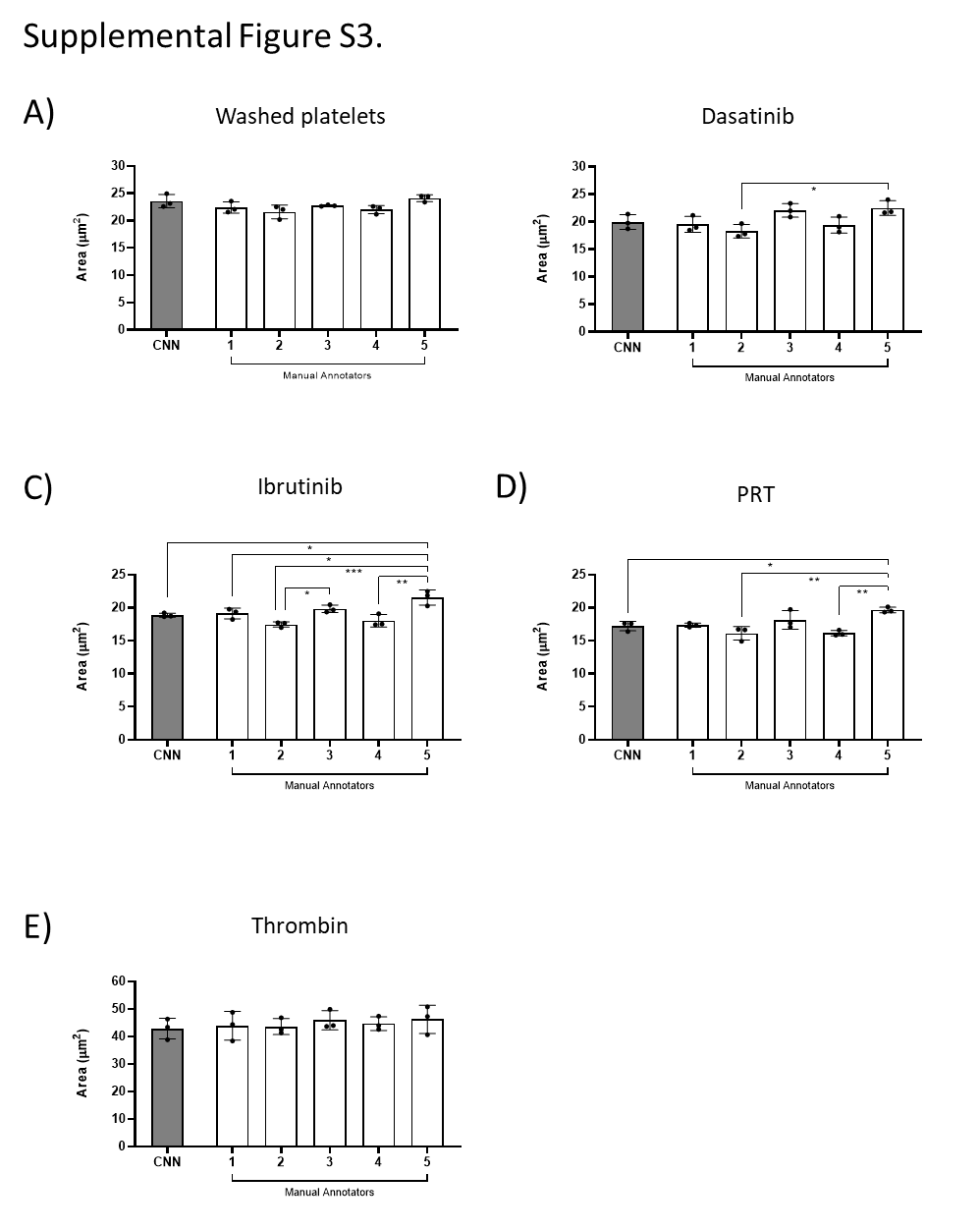
**Supplemental Figure S3. Spread area of platelets treated with inhibitors reveals variation between manual annotators.**

Manual annotations by five annotators were compared to the CNN quantification of spread area of platelets spread over fibrinogen for washed platelets (1 x 10^7^ /mL) (A), platelets treated with either dasatinib [10 μM] (B), ibrutinib [1 μM] (C), PRT-060318 [5 μM] (D), or thrombin [0.1 U/mL] (E). The mean ± SD of three experimental replicates (n=3), whereby each experimental replicate was the mean of three fields of view, were analysed using one-way ANOVA with Bonferroni post-test. *p ≤ .05, **p ≤ .01, ***p ≤ .001, ****p ≤ .0001.


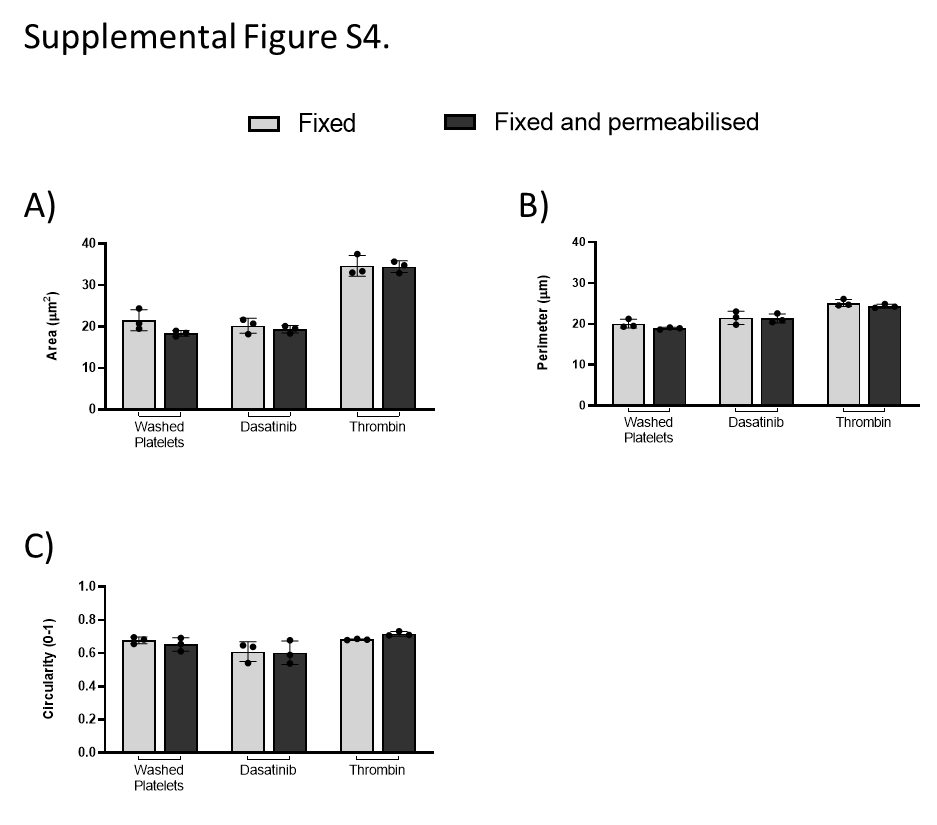


**Supplemental Figure S4. CNN quantification of fixed and permeabilised platelets is the same as fixed platelets.**

Washed platelets (1 x 10^7^ /mL) spread over fibrinogen were either fixed, or fixed and permeabilised, and quantified by the CNN for surface area (A), perimeter (B) and circularity (C) to assess differences between the two methods of sample preparation. The mean ± SD of three experimental replicates (n=3), whereby each experimental replicate was the mean of three fields of view, were analysed using paired two-tailed T-tests. *p ≤ .05, **p ≤ .01, ***p ≤ .001, ****p ≤ .0001.
